# Integrated ensemble of BERT- and feature-based models for authorship attribution in Japanese literary works

**DOI:** 10.3389/frai.2025.1624900

**Published:** 2025-09-22

**Authors:** Taisei Kanda, Mingzhe Jin, Wataru Zaitsu

**Affiliations:** ^1^Graduate School of Culture and Information Science, Doshisha University, Kyoto, Japan; ^2^Research Center for Linguistic Ecology, Doshisha University, Kyoto, Japan; ^3^Institute of Interdisciplinary Research, Kyoto University of Advanced Science, Kyoto, Japan; ^4^Faculty of Psychology, Mejiro University, Tokyo, Japan

**Keywords:** bidirectional encoder representations from transformers, stylometric features, authorship attribution, text classification, integrated ensemble

## Abstract

**Background:**

Traditional authorship attribution (AA) research has primarily relied on statistical analysis and classification based on stylistic features extracted from textual data. Although pre-trained language models like BERT have gained prominence in text classification tasks, their effectiveness in small-sample AA scenarios remains insufficiently explored. A critical unresolved challenge is developing methodologies that effectively integrate BERT with conventional feature-based approaches to advance AA research.

**Revised objective:**

This study aims to substantially enhance performance in small-sample AA tasks through the strategic combination of traditional feature-based methods and contemporary BERT-based approaches. Furthermore, we conduct a comprehensive comparative analysis of the accuracy of BERT models and conventional classifiers while systematically evaluating how individual model characteristics interact within this combination to influence overall classification effectiveness.

**Methods:**

We propose a novel integrated ensemble methodology that combines BERT-based models with feature-based classifiers, benchmarked against conventional ensemble techniques. Experimental validation is conducted using two literary corpora, each consisting of works from 10 distinct authors. The ensemble framework incorporates five BERT variants, three feature types, and two classifier architectures to systematically evaluate model effectiveness.

**Results:**

BERT demonstrated effectiveness in small-sample authorship attribution tasks, surpassing traditional feature-based methods. Both BERT-based and feature-based ensembles outperformed their standalone counterparts, with the integrated ensemble method achieving even higher scores. Notably, the integrated ensemble significantly outperformed the best individual model on Corpus B—which was not included in the pre-training data— improving the F1 score from 0.823 to 0.96. It achieved the highest score among all evaluated approaches, including standalone models and conventional ensemble techniques, with a statistically significant margin (*p* < 0.012, Cohen’s d = 4.939), underscoring the robustness of the result. The pre-training data used in BERT had a significant impact on task performance, emphasizing the need for careful model selection based not only on accuracy but also on model diversity. These findings highlight the importance of pre-training data and model diversity in optimizing language models for ensemble learning, offering valuable insights for authorship attribution research and the broader development of artificial general intelligence systems.

## 1 Introduction

Authorship attribution (AA) entails identifying the author of texts whose authorship is unknown authorship (Stamatatos, 2009; Zheng and Jin, 2022; Xie et al., 2024). Various studies on AA have been conducted, tracing back more than 100 years. Mendenhall (1887) counted the number of letters in each word used in a sentence, analyzed the relative frequency curves, and demonstrated that the curves varied among authors and could be a distinctive characteristic of each author. Mendenhall (1901) also demonstrated that Shakespeare predominantly used four-letter words, whereas Bacon favored three-letter words. This finding challenged the theory that Bacon authored a series of satirical plays under the pseudonym “Shakespeare” to protest against the oppressive government.

In this paper, we refer to datasets related to stylistic analysis—such as word length data—as features. They are distinctive techniques or devices that an author uses to create a particular effect in a text. These are woven throughout the work. Before the 1950s, word, sentence, and paragraph lengths, which were easy to quantify, were mainly used for statistical analysis. With the development of computational environments, many scholars have proposed extracting stylistic features from text based on aspects such as character, word, part-of-speech structure, grammar, and syntax (Yule, 1939; Lagutina et al., 2019; Neal et al., 2017; Zheng and Jin, 2022; Cammarota et al., 2024). Stylistic features that are mechanically aggregated from texts contain a lot of noise.

Stylistic features, subsequently referred to as features in this paper, are language-dependent. For example, Japanese or Chinese differ from Western languages in character forms, writing styles, lack of segmentation, and grammatical structures. One of the most evident ways in which Japanese or Chinese are unique is the character forms and the fact that they are not divided into words. Therefore, the elements that appear as features, such as characters, words, and phrases, also vary depending on the segmentation method.

AA was first performed in stylistic studies of literary works. Over time, it has also been applied to detect fake news, address authorship issues, identify plagiarism, and investigate matters in criminal and civil law (Zaitsu and Jin, 2023; Cammarota et al., 2024; Zaitsu et al., 2024). With the rapid development of computer science, powerful machine learning classifiers and pre-trained language models (PLMs) are being consecutively developed, and the AA environment is changing rapidly.

Several classifiers are now commonly used for text classification, including penalized logistic regression, support vector machine (SVM), random forest (RF), boosting methods (such as AdaBoost, XGBoost), neural network approaches, and PLMs such as bidirectional encoder representations from transformers (BERT) and its derivatives, RoBERTa and DeBERTa. However, because model performance depends heavily on task specifics, sample size, and hyperparameters, identifying the best model and guiding end users on tool adaptation remains challenging.

Against this backdrop, this study focused on significantly improving AA scores in small-scale samples and literary works by employing an integrated ensemble that combines traditional feature- and classifier-based methods with BERT-based methods.

This study addresses the following key research objectives in the context of AA for short literary texts:

(1) To evaluate the effectiveness of BERT-based models in AA tasks involving limited sample sizes.
(2) To examine whether an integrated ensemble of BERT-based and feature-based classifiers can enhance AA accuracy.
(3) To investigate the extent to which the pre-training data of BERT models influences task performance.

## 2 Related research

Since the 1960s, multivariate data analysis methods—using features extracted either manually or automatically from texts—have been applied to AA tasks. These methods include unsupervised techniques, such as principal component analysis, correspondence analysis, and clustering, as well as supervised techniques, such as linear and nonlinear discriminant analysis.

Since the 1990s, neural networks (Kjell, 1994; Hoorn et al., 1999), SVM, RF, boosting classifiers have been employed (Zheng and Jin, 2022; Xie et al., 2024; Jin and Murakami, 2017; Liu and Jin, 2022). Jin and Murakami (2017) demonstrated that RF is more effective than SVM for noisy data. They also analyzed the decline in the RF and SVM scores as sample size decreased.

Liu and Jin (2022) conducted a comparative analysis of genre and author classifications of mixed-genre texts using 14 feature datasets, including character, POS tag, token, and token–POS tag n-grams (*n* = 1–3), as well as phrase patterns and comma positions. Seven classifiers were employed: SVM, RF, AdaBoost, HDDA, LMT, XGBoost, and Lasso. The results indicate that features and classifier scores vary across different cases. In other words, even within the same corpus, variations arise where combinations such as character bigrams with RF yield higher scores, while token unigrams with AdaBoost may perform best.

Regarding the use of features and classifiers in AA, there have been studies on methods for feature extraction and classifier arrangements; however, there has been no significant progress in score improvement. Considering this, Jin (2014) proposed an ensemble approach that employed multiple feature vectors (character bigram, token and POS tag uigram, POS tag bigram, phrase pattern) and classifiers (RF, SVM, LMT, ADD, DWD, HDD). This approach was tested on three text types: novels, student essays, and personal diaries. Four types of features and six classifiers were used to classify the texts. The ensemble of 24 combinations showed strong classification performance and robustness across text types.

Zaitsu and Jin (2023) investigated whether it is possible to distinguish between human-written and ChatGPT-generated (versions 3.5 and 4) Japanese academic paper abstracts using feature-based stylometric analysis. Their findings revealed that Japanese texts produced by ChatGPT exhibit distinct stylistic characteristics that diverge from those found in human-authored writing.

Building on this foundation, Zaitsu et al. (2024) extended their investigation to examine whether fake public comments (approximately 600 characters per text) generated by GPT-3.5 and GPT-4 could be differentiated from genuine human-written comments using stylometric techniques. In this follow-up study, a comprehensive set of stylometric features was employed, including phrase patterns, POS bigrams and trigrams, bigrams of postpositional particles, the positioning of commas, and the use of function words. These features were analyzed using a RF classifier. Although classification performance varied depending on the learning paradigm (i.e., zero-shot vs. one-shot learning), results from 10-fold cross-validation demonstrated that the integrated feature set achieved a mean accuracy of 88.0% (sd = 3.0%) in identifying both the type of large language model (LLM) used and whether the text was human-written. These findings suggest that fake public comments generated by ChatGPT are, to a considerable extent, distinguishable from authentic ones. However, the achieved level of accuracy remains insufficient for practical deployment. To effectively curb the rapid proliferation of AI-generated disinformation and fake news, it is imperative to develop more robust and high-performance detection systems that go beyond stylometric classification alone.

Ensembles of multiple classification models generally match or surpass the best score achieved by any individual model. Moreover, ensembles tend to be more robust than single models. Robustness is crucial for drawing reliable conclusions in real-world applications. As a result, ensemble methods are increasingly being applied in practical settings (Bacciu et al., 2019; Lasotte et al., 2022). Bacciu et al. (2019) performed text classification using a soft-voting ensemble that combined multiple feature sets with a single classifier (SVM). In contrast, Lasotte et al. (2022) employed an ensemble of four classifiers applied to a single feature set to detect fake news, demonstrating the effectiveness of ensemble learning.

In 2019, Google introduced BERT, a model pre-trained on a large English corpus (Wikipedia and BookCorpus), which achieved state-of-the-art (SOTA) performance across several natural language processing (NLP) tasks (Devlin et al., 2019). BERT is based on a transformer architecture and deep neural network, trained on large-scale data to embed words and their contextual relationships into fixed-length vectors. It embeds words and their contextual relations by quantifying them into fixed-length vectors. BERT is available in two configurations: BERT-base with 12 transformer layers and 768 hidden units, and BERT-large with 24 layers and 1,024 hidden units. While BERT-large offers greater capacity for complex NLP tasks, it requires significantly more computational resources. BERT-large has more parameters and can be adapted to more complex NLP tasks. However, it requires more computational resources and time.

BERT’s performance depends heavily on the corpus and language used during pre-training. End users typically fine-tune BERT on task-specific corpora to adapt the model to their needs. Since Devlin et al. (2019), numerous BERT-based models have been proposed and validated across diverse tasks, including question answering, news classification, sentiment analysis, finance, and healthcare. Despite this progress, identifying the most suitable model for a given task remains an open challenge, as performance varies depending on task characteristics and model configurations.

### 2.1 Comparison of PLMs and classifiers

Numerous pre-trained BERT models have been developed, prompting empirical studies aimed at identifying the most suitable model for specific tasks. Qasim et al. (2022) conducted binary fake news classification using two datasets (COVID-19 fake news and extremist–non-extremist datasets) with nine different BERTs. The sample size was over 10,000. Performance varied between BERT and RoBERTa depending on the dataset, and no definitive conclusion could be drawn regarding which model was superior. Similarly, the performance of base and large models varied depending on the dataset, with large models not always scoring higher than their base counterparts.

Karl and Scherp (2023) conducted a comparative analysis of 14 models, including BERT-, BoW-, graph-, and LSTM-based architectures. The SOTA performance depended on the dataset. Notably, even the smallest of the four datasets contained more than 8,000 samples.

Sun et al. (2023) evaluated six and eight PLMs across different benchmark datasets. In the benchmark data used, the SOTA performance of the BERT models was dataset-dependent. Among the seven datasets used, the smallest contained more than 7,000 samples.

Prytula (2024) compared and analyzed the binary classification of positive and negative user comments on a dataset of approximately 11,000 user comments written in Ukrainian, comparing the BERT, DistilBERT, XLM-RoBERTa, and Ukr-RoBERTa models. XLM-RoBERTa achieved the highest accuracy. However, when factoring in training time and overall classification metrics, Ukr-RoBERTa was deemed the most effective.

Abbasi et al. (2022) compared the performance of DistilBERT, BERT, and an ensemble of classifiers using features to determine the authors of news articles. Their results showed that DistilBERT outperformed both BERT and the feature-based ensemble. The study involved classifying 10 or 20 authors using a dataset of 50,000 news articles.

### 2.2 Effect of pre-training data on the BERT model

Mishev et al. (2020) conducted a comparative analysis of 29 models, including both feature-based methods and BERT-based models, for sentiment analysis in finance using two datasets: Financial Phrase-Bank and SemEval-2017 Task 5. The results indicated that FinBERT, pre-trained on Reuters financial data, underperformed compared to BERT models pre-trained on Wikipedia and BookCorpus.

Arslan et al. (2021) used the BBC News and 20News datasets to compare the performance of FinBERT, built from financial datasets, with five general-purpose PLMs (BERT, DistilBERT, RoBERTa, XLM, and XLNet) trained on corpora such as Wikipedia. In addition to generic benchmark datasets, they also used proprietary data obtained from financial technology partners. The results showed that FinBERT did not outperform general-purpose BERT models, even after vocabulary adaptation. More than 2,000 data samples were used, even for small corpora.

Suzuki et al. (2024) developed FinDeBERTaV2 using financial data and compared its performance with GenDeBERTaV2. The results demonstrated that GenDeBERTaV2 excelled in general tasks, while FinDeBERTaV2 showed superior performance in financial applications.

Vanetik et al. (2024) classified the genre of Russian literature using stylistic features and BERT. The results indicated that the classifier using stylistic features outperformed BERT, with higher accuracy scores. Experimental results also showed that ruBERT, pre-trained on a large Russian corpus, underperformed compared to the multilingual BERT model. The SONATA dataset used was a random sample of 11 genres, with 100 samples per genre, and the text chunks were extracted manually.

These findings highlight inconsistencies in how pre-training corpora affect task performance, underscoring the need for continued investigation. Kanda and Jin (2024) conducted a comparative analysis of the effects of pre-training data on tasks using literary works for four different types of BERT, including one trained on literary texts. Their study, reported in Japanese, confirmed that pre-training data had a significant impact on task outcomes. Additional experiments and analyses are presented in Subsection.

### 2.3 Ensemble of BERTs

An ensemble of BERT models was also discussed by Devlin et al. (2019), the original paper that introduced BERT. Ensembling BERTs involves (1) combining results from different models obtained by varying fine-tuning datasets or training configurations, and (2) aggregating outputs from multiple BERT models created with different pre-training data and parameters.

Tanaka et al. (2020) conducted a study using BERT for Japanese texts longer than 510 tokens. They extracted 510-token segments by shifting the window at regular intervals toward the end of the text to create multiple samples. Subsequently, they ensembled the BERT results obtained from these samples. The ensemble outperformed the single BERT model using only one 510-token segment. In their experiment, they used three datasets, each with 2,000 samples, for training and testing.

Dang et al. (2020) won the SMM4H Task1 competition with an ensemble of 20 results obtained from 10 sets of samples across 10 cross-validations and two different BERT models.

Abburi et al. (2023) won first prize in the 2023 competition for their study on identifying LLM-generated texts using an ensemble of DeBERTa, XLM-RoBERTa, RoBERTa, and BERT. The ensemble used a classifier that took as input a vector composed of outputs from the BERT-based models. The data used came from four datasets distributed in the competition, with the smallest sample size exceeding 20,000.

### 2.4 Ensemble of BERT and features

Tanaka et al. (2020) demonstrated that text classification using a three-layer neural network on data composed of Bag-of-Words (BoW) vectors concatenated with BERT embedding vectors improved classification scores. For 510-token inputs, the score increased by 2.1 points.

Fabien et al. (2020) applied logistic regression to the classification outputs of a single BERT model, stylistic features, and sentence structure features. However, the ensemble scores were generally lower than those of the single BERT model, possibly due to feature redundancy or model incompatibility. Their experiment used four datasets, and even in the case with the fewest samples per author, the count exceeded 900.

Wu et al. (2021) won the competition by combining the outputs of BERT and RoBERTa with those from a tree-based model, applied to a disease-related question-and-answer dataset. The ensemble’s F1 score was 3.38 points higher than that of the best-performing individual model, RoBERTa_wwm. The training dataset contained 30,000 samples.

Strøm (2021) detected shifts in authorial style by stacking ensembles built from 478-dimensional stylistic features and 768-dimensional BERT embedding vectors. Their system achieved the highest score in the classification task of that year’s competition. However, the score improvements over the LightGBM classifier were modest: 1.93, 2.86, and 1.01 points across three tasks. The dataset used contained over 10,000 samples.

Based on the aforementioned related studies, the research and application of AA reveal the following challenges.

(1) Sample issue

The BERT-related studies cited above show that the datasets used for evaluating or applying BERT models typically contain thousands of samples. For example, Devlin et al. (2019) reported that the smallest dataset in the GLUE benchmark contained 2.5 K samples. While large datasets are common in large-scale classification systems, real-world forensic problems—such as those involving criminal or civil law—often rely on far fewer samples, sometimes fewer than 10. This highlights the need for further research on small-sample scenarios. However, no existing studies have directly addressed this challenge.

(2) BERT and challenges related to pre-trained data

Although many pre-trained models have been proposed, no consensus has been reached regarding which model performs best across tasks. This is because model performance depends on factors such as evaluation sample size, task type, and tuning parameters. Therefore, further research is needed to guide end users in selecting appropriate models and leveraging existing data effectively when applying BERT. Several studies have examined how pre-training data influences downstream task performance; however, no clear conclusions have been drawn. Moreover, the impact of pre-training data on tasks involving literary texts remains unexplored.

(3) Ensemble issue

We found no studies that combined outputs from multiple BERT models trained on different pre-training corpora with results from multiple feature datasets and classifiers. In most of the above studies, features were concatenated into a single vector. Unlike topic classification, several stylistic features have been proposed for AA; however, identifying the most effective features remains challenging, as they depend heavily on the writer’s genre and style. Additionally, although high-dimensional data are easy to collect, merging them into a single dataset can significantly affect the performance of classifiers. For example, aggregating character bigrams can result in thousands of dimensions. Using ultra-high-dimensional data—formed by horizontally combining multiple feature sets with thousands of dimensions—is not advisable when working with small datasets containing only tens or hundreds of samples. While some studies have explored how to utilize the many available variations of BERT and feature-based models, they have yielded only marginal improvements with limited practical impact.

Building on the aforementioned challenges, this study presents an empirical analysis to evaluate whether an integrated ensemble of BERT-based and feature-based models can significantly enhance AA in literary texts, particularly for small samples of approximately 500 tokens. The analysis also examines the impact of BERT’s pre-training data and the contribution of individual models within the ensemble.

## 3 Experiments

**Figure 1:**
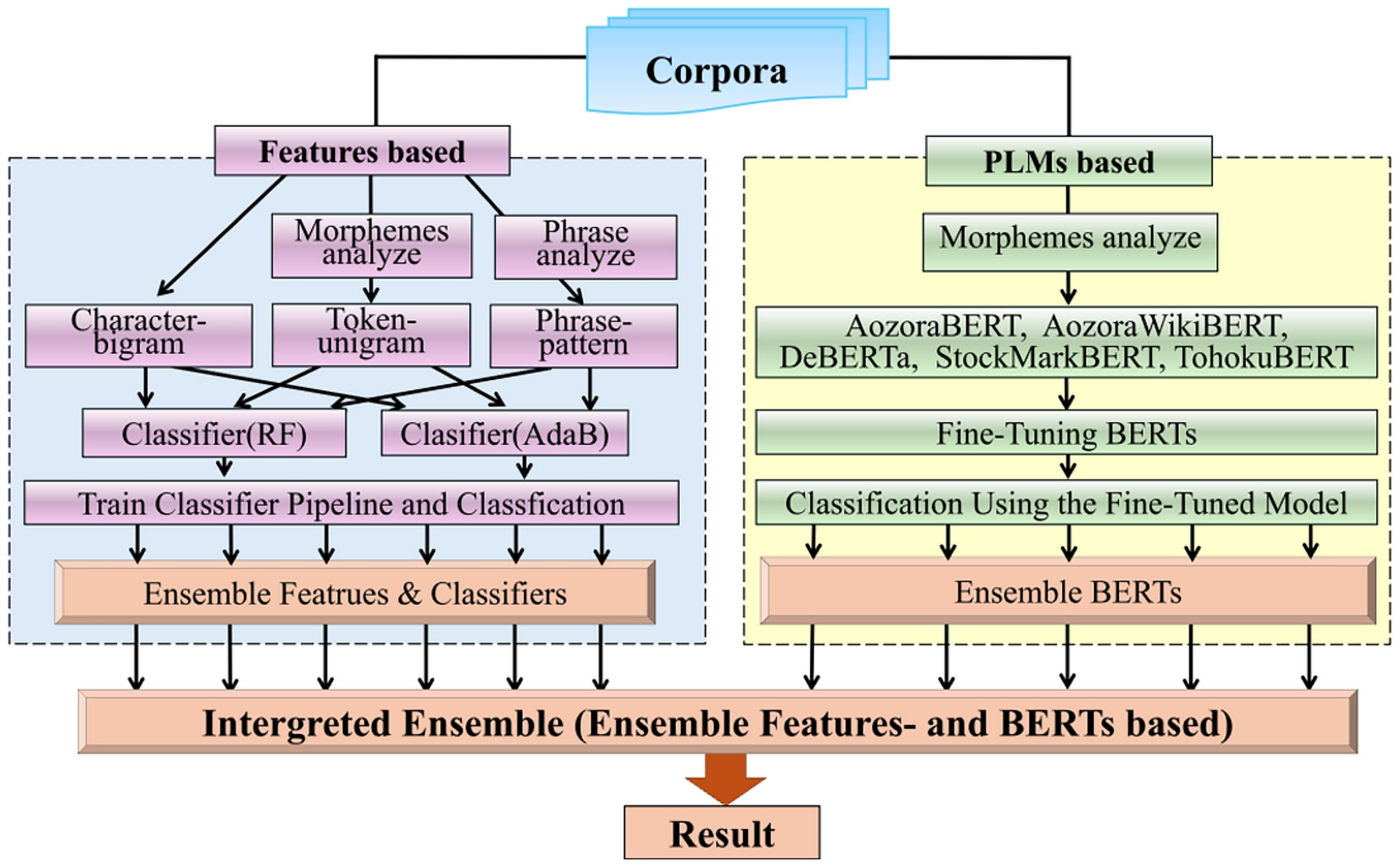
The overview of workflow of this study.

We classified two corpora using multiple models composed of BERTs and features-based classifiers; then, we applied an integrated ensemble of BERT- and feature-based models to the classification results. The workflow of this study experiment is shown in Figure 1.

The basic concept underlying these ensembles is collective intelligence. Since the 1990s, ensemble learning has been used in the field of machine learning to improve performance by combining the results of multiple models. Ensembles include voting and stacking methods; however, voting methods are most often used to integrate the predictions of base models. There are two types of voting: hard and soft. Hard voting, also known as majority voting, involves aggregating the prediction results of each model and selecting the class with the most votes as the final prediction. Soft voting involves summing or weighting the probability scores output by each model and using the highest score as the final prediction. In this study, we used an ensemble method based on soft voting. Specifically, the probability vectors, *Model^K^(x)*, obtained from different models (classifiers or BERT) are summed or averaged as shown in Equation 1, and the author with the largest value is assigned as the author of index *j*∗. In the equation, *w_k_* is the weight of classification model *k*. We used a vector of 1 when not considering weights, and the F1 score of each model when weights were considered.

(1) $$j^{*}=\underset{j}{\text{argmax}}[\frac{1}{K}\sum_{k=1}^{K}w_{k}\text{Mode}l^{k}(X)]\;$$

In this study, the results of individual classifiers were probability vectors, and the results of each BERT were converted into probability vectors using a Softmax function.

### 3.1 Features and classifiers used

Several features have been proposed for the quantitative analysis of writing style and author estimation. We used character-bigram, token-unigram, and phrase pattern (Bunsetsu-pattern) as representative stylistic features, as demonstrated in previous studies (Jin and Murakami, 2017; Liu and Jin, 2022; Zaitsu and Jin, 2023). These features, including character bigrams, token unigrams, and phrase patterns, are based on fundamental Japanese linguistic units: characters, words, and phrases (Bunsetsus). We intentionally limited our feature set to these three types to ensure a balanced comparison with the number of BERT models, despite the possibility of incorporating additional features.

(1) Character-bigram

Character n-gram is a dataset that aggregates the patterns of *n* adjacent characters. In this study, to estimate authorship using short sentences of 510 tokens (approximately 800 characters), we used the most frequently used characters bigram based on the dimensionality and sparsity of the data.

(2) Token-unigram

Morphemes were analyzed using the MeCab^1^ and UniDic^2^ dictionaries. Among the n-grams of token, we used the unigram, which mainly reflects the characteristics of an author.

(3) Phrase pattern

Many indicators of authorship are also found in the sentence syntax. The basic unit of Japanese parsing is a phrase (*Bunsetsu*). Jin (2013) proposed and validated a method that patterned information in a phrase. To illustrate for Japanese, consider a sentence, “BERTは分類に有効である。” (BERT is effective for classification). The sentence is split into phrases as “BERT (noun)は (particle)/分類 (noun)に (particle)/有効 (adjectivalNoun)で (adjective) ある (verb)。 (punctuation) “. (BERT is/ effective for/ classification). In this context, the symbol “/” indicates the phrase boundaries, and the string within the parentheses represents the POS tag of the preceding morpheme. The first phrase in the sentence, “BERT (noun)は (particle) “consists of a noun “BERT” and a particle “は.” The word “BERT” is content-dependent; therefore, if it is used as a feature in the AA, it will be a noise in the analysis of the author’s characteristics. Therefore, the POS tag noun is used in BERT to mask the content word. The result, “BERT (noun)は (particle) “, is patterned after “noun + は(particle) “. The particle “は” may change depending on the author. This is because using “が “(ga) instead of “は” (wa) does not pose a grammatical problem. Words that are not characteristic of the writer are masked with their POS tags in phrase patterns. The data related to phrase patterns is an aggregation of such patterns categorized by type. In Japanese, the usage data of particles and punctuation have been demonstrated to be effective features for AA in numerous studies (Jin and Zheng, 2020). The phrase pattern can capture individual habits, specifically how distinctive elements of a writer’s style are combined and used. Although a phrase pattern never scores high in author estimation, it is robust to text content and genre because content words are masked by their POS tags. In a study on AA for texts containing mixed genres (Liu and Jin, 2022), the combination of phrase pattern features and a Lasso classifier achieved the highest accuracy (0.895) among 14 feature types and seven classifiers tested in the comparative analysis. In this experiment, CaboCha^3^ was used for the phrase segmentation process. Morphemes were masked using POS tags—excluding particles, punctuation marks, conjunctions, and adjectives—based on the syntactic parsing results generated by CaboCha. The following presents the core part of the pseudocode for processing bunsetsu patterns (Algorithm 1).

(4) Classifiers

**ALGORITHM 1:**
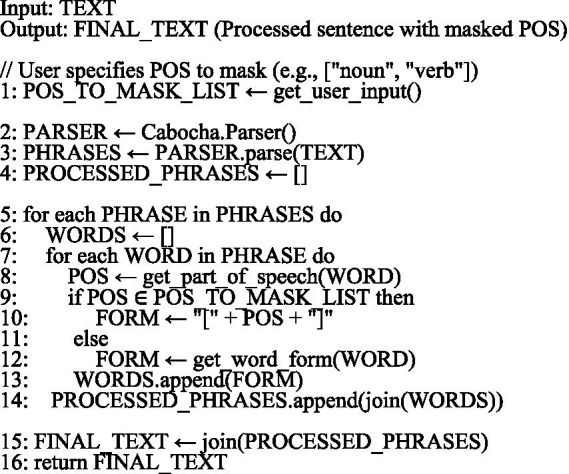
Core pseudocode for parser (Bunsetsu) pattern generation.

We used two types of classifiers, Random Forest (RF) and AdaBoost (Ada), which have been reported to achieve relatively high performance. For RF, we used the default settings of the randomForest (ntree = 500, maxnodes = NULL, …) function in the R package randomForest. This function is based on the algorithm proposed by Breiman (2001). However, although XGBoost is reported to have excellent performance among boosting algorithms, our study, which focused on using stylistic features, did not obtain any results that demonstrated XGBoost’s superior performance over AdaBoost (Liu and Jin, 2022). For AdaBoost, we used the default settings of the boosting (boos = TRUE, mfinal = 100, coeflearn = ‘Breiman’…,) function in the R package adabag. This function is based on the algorithm AdaBoost.M1 (Alfaro et al., 2013).

### 3.2 Selected PLMs

In this study, we used several BERT models as pre-trained language models (PLMs). BERT is a PLM built on the Transformer architecture, taking a fundamentally different approach from traditional classifiers. Traditional classifiers rely on extracting features from text, representing them as vectors, and applying models such as logistic regression. These methods focus solely on frequency-based feature extraction, ignoring sequential and contextual information.

BERT generates contextualized word embeddings using a multi-layer Transformer pre-trained on large text corpora. Its self-attention mechanism analyzes token relationships bidirectionally, enabling strong context modeling and handling long-range dependencies. Pre-training involves Masked Language Modeling (MLM) and Next Sentence Prediction (NSP), allowing BERT to learn deep linguistic patterns from unlabeled data. Fine-tuning adapts BERT for tasks like text classification, achieving high accuracy even with limited labeled data through transfer learning.

We used BERT models pre-trained on Japanese corpora, which are described below. The multilingual XLM-RoBERTa has been released as an advanced variant of BERT. XLM-RoBERTa uses SentencePiece, a tokenizer designed to maintain consistency across languages, but it does not fully leverage the linguistic characteristics of Japanese. Additionally, the accuracy of SentencePiece has been reported to be lower than that of the Japanese-specific tokenizer MeCab. Therefore, this study employed a BERT model specialized for the Japanese language.

The following five BERT models were selected based on the diversity of the pre-training data used for training and their performance. In addition, we are limited to BERTs trained using pre-training data processed with tokenizers that are highly rated against the Japanese language.

(1) Japanese BERT trained on Wikipedia^4^

There are many BERTs pre-trained on the Japanese Wikipedia. Based on preliminary analysis, we used the most widely used basic version published by Tohoku University that base version of a publicly available model pre-trained on data tokenized using MeCab and WordPiece.

There are many BERT models pre-trained on the Japanese Wikipedia. Based on preliminary analysis, we adopted the most widely used basic version — published by Tohoku University as the foundation of a publicly available model — which was pre-trained on data tokenized with both MeCab and WordPiecev.

(2) Japanese BERT trained on Aozora Bunko^5^

Koichi Yasuoka has released a pre-trained BERT model trained on the Aozora Bunko corpus — a public-domain repository of Japanese literary works. From the published variants, we adopted the model pre-trained using MeCab tokenization with the UniDic dictionary.

(3) Japanese BERT trained on Aozora Bunko and Wikipedia (see text footnote 5, respectively).

Koichi Yasuoka has released BERT models pre-trained on a combined corpus of Aozora Bunko and Wikipedia. From these, we adopted a model pre-trained on data tokenized using MeCab with the UniDic dictionary.

(4) DeBERTa^6^

He et al. (2021) proposed DeBERTa, an improved version of the BERT and RoBERTa models. DeBERTa introduces Disentangled Attention, a mechanism that separately encodes word content and positional information into distinct vectors. This separation enhances contextual representation learning, leading to superior performance over standard BERT architectures. Furthermore, DeBERTa employs an Enhanced Mask Decoder during pre-training, which explicitly incorporates absolute positional data to better align contextual and positional features. The objective of this design is to improve the prediction accuracy of masked tokens compared to other conventional BERT models.

We used the Japanese DeBERTa V2-base model pre-trained on Wikipedia, CC-100, and OSCAR corpora with JUMAN++ tokenization.

(5) BERT StockMark^7^

StockMark Inc. released a BERT model pre-trained on Japanese news articles. As new words are created annually in business news, unknown words are processed as [UNK] tokens without subword settings. We used a model pre-trained on data tokenized using MeCab with the NEologd dictionary.

For brevity, we used the abbreviations TohokuBERT (T), AozoraBERT (A), AozoraWikiBERT (AW), DeBERTa (De), and StockMarkBERT (S) for the BERTs.

### 3.3 Used corpora

Two corpora were used in this study: Corpora A and B. Corpus A consisted of 10 authors selected from the literary authors available in the Aozora Bunko, and 20 works for each author. Corpus A data made up approximately 0.03% of the pre-training data in Aozora Bunko. Specifically, the corpus contained 20 works by Akutagawa Ryunosuke (1892–1927), Izumi Kyoka (1873–1939), Kikuchi Kan (1888–1948), Mori Ogai (1862–1922), Natsume Soseki (1867–1916), Sasaki Ajitsuzo (1896–1934), Shimazaki Toson (1872–1943), Dazai Osamu (1909–1948), Okazaki Kido (1872–1939), and Umino Juzo (1897–1949). The works were prioritized by those that had already been converted to the new script and new kana usage and those that were published in the same year.

Corpus B was an electronic version of 20 works in paper form by 10 writers who are still active in the field and were not used for the pre-training of BERT. These authors were Suzuki Kouji (1957-), Kishi Yusuke (1959-), Shuichi Yoshida (1968-), Miyabe Miyuki (1960-), Morimi Tomihiko (1979-), Ishida Ira (1960-), Murakami Haruki (1949-), Murakami Ryu (1976-), Higashino Keigo (1958-) and Minato Kanae (1973-).

We conducted experiments to attribute the works of 10 authors each using these two corpora of literary works. The total number of tokens used was 101,167 for Corpus A and 101,165 for Corpus B.

### 3.4 Experimental setup and metrics for evaluating results

Following previous research, we used the five-fold cross-validation. Typically, k-fold cross-validation involves randomly dividing the data into k subsets. However, owing to the small sample size of our data, random division may bias the balance of class labels. Therefore, in this experiment, we used a stratified sampling method to divide the dataset into five folds in the following proportions: training data (160), validation data (20), and test data (20) per fold. The number of works by each author for learning and testing in all the folders was designed to ensure balance.

In languages where texts are not inherently segmented into tokens, such as Japanese and Chinese, processing with BERT necessitates tokenization at either the character or morpheme level. Although character-level BERT models for Japanese demonstrate suboptimal performance, current implementations preprocess texts via morphological analysis, treating each morpheme as a discrete token. In this study, we morphologically analyzed 200 works each from Corpus A and Corpus B using MeCab for morphological segmentation. Each morpheme was treated as a single token, with the first 510 tokens extracted and formatted as input text for BERT. To meet BERT’s maximum input length of 512 tokens, [CLS] and [SEP] tokens were prepended and appended, respectively, during the conversion of tokenized sequences into input IDs. BERT then converted the input into vector embeddings through deep learning and output classification results based on those vectors. In this study, for fairness, only the first 510 tokens were used for all processes, including feature-based methods.

**Figure 2:**
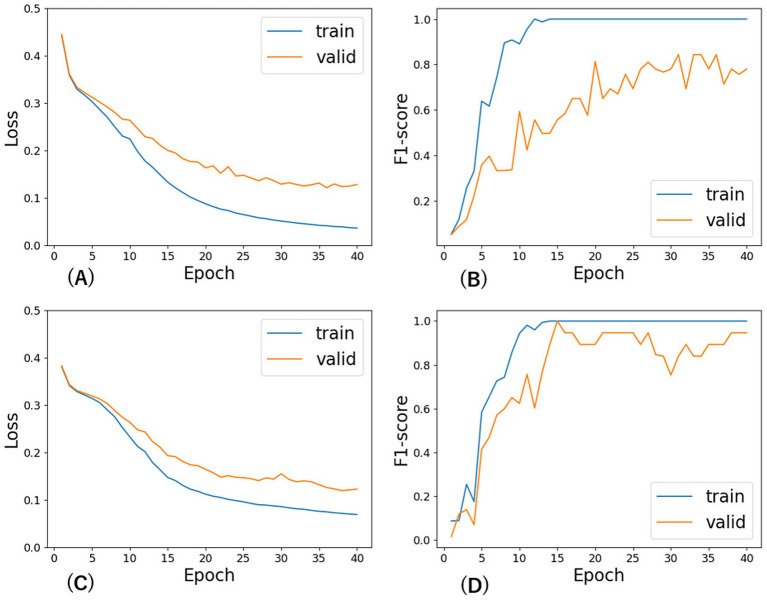
Learning curves of F1 score and loss across epochs for TohokuBERT and DeBERTa on CorpusB. **(A)** Loss vs. epochs for TohokuBERT, **(B)** F1 score vs. epochs for TohokuBERT, **(C)** Loss vs. epochs for DeBERTa, **(D)** F1 score vs. epochs for DeBERTa.

Based on a preliminary analysis of the hyperparameters for fine-tuning BERT, we set the mini-batch size to 16 and learning rate to 2e-05 for any BERT and used AdamW as the optimization algorithm. The relatively small size of our training dataset necessitated a greater number of epochs compared to previous studies that used large-scale corpora. Although the number of epochs required for loss convergence varied slightly among BERT-based models, the overall trend was consistent. As shown in Figure 2, which presents the learning curves for TohokuBERT and DeBERTa on both the training and validation sets of CorpusB, the validation F₁ score began to stabilize around epoch 35. Although extended training would have been preferable based on the trend of the validation loss, the number of epochs was uniformly set to 40 for all models due to operational constraints. While extensive hyperparameter tuning was beyond the scope of this study, preliminary experiments confirmed that the chosen configuration (batch size = 16, learning rate = 2 × 10^−5^, epochs = 40) yielded consistent results across all models. Although the number of epochs required for loss convergence varied slightly among BERT-based models, the overall trend was consistent. Early stopping was not applied.

The most widely used performance measure of a model is the macro average of the F1 measure, which balances the recall and precision measures. The equations for each evaluation index are as follows. The estimation results for author *i* (*i* = 1, 2, 3…, *M*) and other authors are shown in the confusion table in Table 1.

(2) $${\text{Recall}}_{i}=\frac{1}{M}\sum_{i=1}^{M}(\frac{{\mathrm{TP}}_{i}}{{\mathrm{TP}}_{i}+{\mathrm{FN}}_{i}})$$

(3) $${\text{Precision}}_{i}=\frac{1}{M}\sum_{i=1}^{M}(\frac{{\mathrm{TP}}_{i}}{{\mathrm{TP}}_{i}+{\mathrm{FP}}_{i}})$$

(4) $$\begin{array}{c} F1_{i}=\frac{2({\text{Precision}}_{i}\times {\text{Recall}}_{i})}{{\text{Precision}}_{i}+{\text{Recall}}_{i}} \end{array}$$

(5) $$\begin{array}{c} \text{Macro}\;F1=\frac{1}{M}\sum_{i=1}^{M}F1_{i} \end{array}$$

**Table 1:** Confusion table of classification results.

For author *i*		Model output	
		*P*	*N*
Real data	*P*	*TP_i_*	*FN_i_*
	*N*	*FP_i_*	*TN_i_*

The ${\text{Recall}}_{i}$ , ${\text{Precision}}_{i}$ , and $F1_{i}$ mentioned above were the metrics used to evaluate the performance of the classification model results for each individual class (author) *i*. *M* is the number of classes (authors). ${\text{Recall}}_{i}$ (True Positive Rate) is the proportion of actual positives for class *i* correctly identified by the model. ${\text{Precision}}_{i}$ is the proportion of predicted positives for class *i* that are true positives. $F1_{i}$ is the harmonic mean of ${\text{Precision}}_{i}$ and ${\text{Recall}}_{i}$ , providing a balanced measure of the model’s performance for class *i*. Hereafter, Macro F1 is abbreviated as F1.

## 4 Results and analysis

### 4.1 BERT results

We evaluated the performance of the BERT models on the test data at the point when performance plateaued on the validation data. Table 2 presents the experimental results of BERT on the corpora. The highest F1 scores for both corpora are shown in bold. For Corpus A, Model AW, pre-trained on Wikipedia and Aozora Bunko, achieved the highest score and the smallest standard deviation, followed by Model A, which was pre-trained on Aozora Bunko. The F1 scores of both models were more than 30 points higher than that of Model T, which was trained using only Wikipedia. This result likely reflects the fact that Corpus A was included in the pre-training data.^8^

**Table 2:** Results of discrimination of 10 authors by BERT.

Corpus	BERT	Recall	Precision	F1
Corpus A	TohokuBERT(T)	0.653 ± 0.209	0.640 ± 0.201	0.642 ± 0.201
	AozoraBERT(A)	0.973 ± 0.044	0.970 ± 0.067	0.969 ± 0.037
	AozoraWikiBERT(AW)	0.973 ± 0.044	0.970 ± 0.048	**0.970 ± 0.026**
	DeBERTa(De)	0.752 ± 0.215	0.680 ± 0.162	0.691 ± 0.153
	StockMarkBERT(S)	0.619 ± 0.210	0.600 ± 0.211	0.600 ± 0.187
Corpus B	TohokuBERT(T)	0.762 ± 0.151	0.740 ± 0.117	0.744 ± 0.120
	AozoraBERT(A)	0.813 ± 0.210	0.770 ± 0.195	0.773 ± 0.167
	AozoraWikiBERT(AW)	0.838 ± 0.116	0.820 ± 0.114	0.820 ± 0.074
	DeBERTa(De)	0.834 ± 0.118	0.820 ± 0.063	**0.823 ± 0.070**
	StockMarkBERT(S)	0.706 ± 0.126	0.690 ± 0.110	0.692 ± 0.099

Bold values indicate the maximum for each corpus.

In Corpus B, Model De achieved the highest F1 score, followed by AW and A. The model with the lowest score in both corpora was S, which had been pre-trained on business news articles. The fact that Model De achieved the highest score for Corpus B suggests that Corpus B was not included in its pre-training data, and that Model De is more generic. The scores of Models AW and A were higher than those of the models pre-trained on Wikipedia and news articles, likely because Corpus B is also a literary work. We believe that this performance difference is due to the influence of text style.

The scores of Model S, pre-trained on Wikipedia and Japanese business news articles, were higher for Corpus B than for Corpus A. This variation may be attributed to differences in vocabulary and grammar characteristic of the period, as most of the works in Corpus B were published in the 1990s. These results suggest that pre-training data influence performance on individual tasks. Generally, BERT performs better when the training data are large and drawn from diverse domains; a similar trend was observed in this study.

### 4.2 Features and classifier results

Experiments on feature extraction from literary works and author identification using classifiers were conducted under the same conditions as those in the BERT experiment, including the length of the works used. The dimensions of the extracted feature datasets were 4,444 for char-bigram, 3,300 for token-unigram, and 804 for phrase pattern. The classification results of the AdaBoost and Random Forest classifiers for these feature datasets are listed in Table 3. Feature extraction and processing in R were performed using MTMineR (Jin and Zheng, 2020).^9^ The average scores for both corpora did not differ significantly. The highest F1 score was achieved by Random Forest using token-unigram features, while the lowest was obtained by Random Forest using phrase pattern features.

**Table 3:** Results of discrimination of 10 authors by features and classifiers.

Corpus	Classifiers	Features	Recall	Precision	F1
Corpus A	AdaBoost	Char-bigram	0.786 ± 0.188	0.760 ± 0.143	0.766 ± 0.152
		Token-unigram	0.767 ± 0.149	0.750 ± 0.097	0.754 ± 0.109
		Phrase pattern	0.762 ± 0.116	0.750 ± 0.165	0.747 ± 0.125
	RandomForest	Char-bigram	0.792 ± 0.130	0.790 ± 0.179	0.784 ± 0.134
		Token-unigram	0.823 ± 0.124	0.810 ± 0.120	**0.810 ± 0.094**
		Phrase pattern	0.714 ± 0.120	0.710 ± 0.185	0.704 ± 0.135
Corpus B	AdaBoost	Char-bigram	0.779 ± 0.125	0.760 ± 0.117	0.761 ± 0.083
		Token-unigram	0.772 ± 0.122	0.760 ± 0.079	0.762 ± 0.091
		Phrase pattern	0.654 ± 0.131	0.650 ± 0.158	0.647 ± 0.131
	RandomForest	Char-bigram	0.780 ± 0.106	0.780 ± 0.215	0.767 ± 0.155
		Token-unigram	0.810 ± 0.109	0.800 ± 0.105	**0.800 ± 0.090**
		Phrase pattern	0.668 ± 0.150	0.650 ± 0.178	0.643 ± 0.142

The values in the table represent the mean ± standard deviation from five-fold cross-validation.

### 4.3 Ensemble of BERTs

There were 26 possible combinations of two or more of the five BERT models. For the weighted ensemble, we used the F1 scores of each BERT model. To save space, summary statistics of the F1 scores for ensembles from both methods are presented in the second and third rows of Table 4. For Corpus A, the maximum ensemble score and the mean increased by 2.0 and 13.7 points, respectively. For Corpus B, the maximum score and the mean increased by 7.9 and 9.2 points, respectively. The weighted ensembles did not show any improvement in score compared to the unweighted ensembles in either corpus. Direct comparison with existing AA methods is challenging due to their reliance on large, publicly available datasets, primarily in English. To enable a meaningful comparison, we adapted established, reproducible methods to our corpus. The results are shown in Table 4.

**Table 4:** Statistics of F1 values for BERT-based and feature-based ensembles and integrated ensembles.

Method	Corpus A		Corpus B	
	mean ± sd	max	mean ± sd	max
A: BERTs	0.775 ± 0.181	0.970	0.770 ± 0.055	0.823
B: Ensemble BERTs (Kanda and Jin, 2024)	0.911 ± 0.091	0.990	0.861 ± 0.030	0.902
C: Weighted Ensemble BERTs	0.910 ± 0.096	0.980	0.861 ± 0.029	0.899
D: Features and Classifiers	0.761 ± 0.036	0.810	0.730 ± 0.067	0.800
E: Ensemble Features and Classifiers (Jin, 2014)	0.852 ± 0.033	0.912	0.817 ± 0.039	0.889
F: Weighted Ensemble of Features and Classifiers	0.851 ± 0.034	0.912	0.828 ± 0.033	0.889
G: Ensemble One Feature and Classifiers and BERTs (Strøm, 2021)	0.934 ± 0.040	0.970	0.887 ± 0.044	0.920
H: Ensemble One BETR and Features and Classifiers (Wu et al., 2021; Abbasi et al., 2022)	0.834 ± 0.127	0.990	0.814 ± 0.052	0.901
I: Integrated Ensemble (proposed method)	0.991 ± 0.003	**1.000**	0.957 ± 0.005	**0.960**
J: Integrated Weighted Ensemble (proposed method)	1.000 ± 0.000	**1.000**	0.953 ± 0.005	**0.960**

The mean and standard deviation (sd) of the top 50 are used for methods with F1 values greater than 50. The values represent the mean, sd, max obtained using the method indicated at the beginning of each row, applied to both corpora.

To account for the combinatorial possibilities in ensemble construction, the F1 scores of the top 10 ensemble sets are listed on the left side of Table 5. For Corpus A, the ensembles {A, S}, {T, A}, {A, AW}, {A, De}, {A, AW, De}, and {AW, S} exceeded the maximum F1 score of any single BERT model. In Corpus B, 22 ensembles surpassed the maximum value of 0.820 achieved by a single BERT. The highest scores were obtained by {T, A, AW, De}, followed by {A, AW, De, S}, {T, A, De, S}, and {T, A, AW, De, S}.

**Table 5:** Top 10 F1 scores for ensemble and integrative ensemble results.

Corpus	BERTs		Features and Classifiers		Integrated Ensemble (Proposed Method)	
	Ensemble Labels	F1	Ensemble Labels	F1	Ensemble Labels	F1
Corpus A	{A, S}	0.990	{1, 3, 5}	0.912	{A, S | 3,5}	1.000
	{T, A}	0.980	{1, 3, 4, 5}	0.912	{A, S | 3,6}	1.000
	{A, AW}	0.980	{1, 3, 4, 5, 6}	0.912	{A, S | 4, 6}	1.000
	{A, De}	0.980	{1, 3, 5, 6}	0.901	{A, S | 5, 6}	1.000
	{AW, S}	0.980	{1, 3, 4}	0.893	{A, S | 3, 5, 6}	1.000
	{A, AW, De}	0.980	{1, 2, 3, 6}	0.883	{A, S | 4, 5, 6}	1.000
	{T, A, AW}	0.970	{1, 2, 3, 4, 6}	0.883	{T, A | 3, 6}	0.990
	{A, AW, S}	0.970	{1, 2, 3, 5, 6}	0.883	{T, A | 1, 3, 6}	0.990
	{T, A, AW, De}	0.970	{1, 2, 3, 4, 5, 6}	0.883	{T, A | 3, 4, 6}	0.990
	{T, A, AW, S}	0.970	{1, 3, 6}	0.882	{T, A | 3, 5, 6}	0.990
	All Models	0.940	All Models	0.883	All Models	0.990
Corpus B	{T, A, AW, De}	0.902	{1, 2, 6}	0.889	{T, De | 1, 2}	0.960
	{A, AW, De, S}	0.901	{1, 2, 4, 6}	0.889	{T, AW | 1, 2}	0.960
	{T, A, De, S}	0.894	{1, 2, 5, 6}	0.889	{T, AW | 1, 2, 4}	0.960
	{T, A, AW, De, S}	0.891	{1, 2, 4, 5, 6}	0.889	{T, AW, De | 1, 2, 4, 6}	0.960
	{T, AW, De, S}	0.89	{1, 2, 4}	0.869	{T, AW, De | 1, 2, 4, 5, 6}	0.960
	{T, A, AW}	0.882	{4, 5, 6}	0.866	{AW, De, S | 1, 2}	0.960
	{T, AW, De}	0.882	{1, 4, 5, 6}	0.859	{AW, De, S | 1, 6}	0.960
	{T, A, AW, S}	0.881	{1, 2, 5}	0.858	{AW, De, S | 1, 2, 4}	0.960
	{A, AW, De}	0.88	{1, 2, 4, 5}	0.858	{AW, De, S | 1, 2, 5}	0.960
	{AW, De}	0.88	{1, 2, 3, 4}	0.855	{AW, De, S | 1, 2, 6}	0.960
	All Models	0.891	All Models	0.855	All Models	0.950

1: Ada + Char, 2: Ada + Token, 3: Ada + Phrase, 4: RF + Char, 5: RF + Token, and 6: RF + Phrase, A: AozoraB, AW: AozoraWikiB, De: DeBERTa, S: StockMarkB, T: TohokuB.

Interestingly, the ensemble with the highest F1 score in both corpora included Model S, despite it having the lowest individual score among all models. Model S was pre-trained on news articles, which differ significantly from the literary style of the target texts. This suggests that while ranking models by individual performance is useful for ensemble selection, incorporating heterogeneous models can also be beneficial.

For both corpora, the ensemble scores of {T, A} were 0.980 and 0.856, respectively, which are higher than those of Model AW (0.970 and 0.820, respectively). Wikipedia and Aozora Bunko were used for pre-training Models T and A, respectively, while both sources were used for Model AW. Nevertheless, the ensemble scores of Models T and A were higher than those of Model AW. This again suggests that model performance is influenced by the pre-training data and other model-specific properties.

### 4.4 Ensemble results for features and classifiers

The ensemble of the six results (1: Ada + Char, 2: Ada + Token, 3: Ada + Phrase, 4: RF + Char, 5: RF + Token, and 6: RF + Phrase) of the two classifiers (Ada and RF) with three features (char-bigram, token-unigram, and phrase pattern) yielded 57 results.

The summary statistics of the ensemble results for both corpora are presented in Table 4. For both corpora, the maximum F1 scores of the ensembles were significantly higher than those of the stand-alone features and classifiers. For Corpus A, the maximum F1 score was 10.1 points higher than those of the single features and classifiers, and the average score was 9.1 points higher. For Corpus B, the maximum F1 score was 8.9 points higher than those of the single features and classifiers, and the average score was 8.7 points higher. Table 5 shows the top 10 scoring ensembles. Some combinations of features and classifiers in the ensemble included Labels 3(Ada + Phrase r) and 6(RF + Phrase), which had the lowest scores. The reasons for this will be analyzed considering the results of the integrated ensemble.

### 4.5 Integrated ensembles

In the integrated ensemble, we combine results from both BERT- and feature-based models. For the five BERT models, the number of combinations involving two or more models is 26. For the feature-based models, there are six results, and the number of combinations involving two or more of them is 57. Since the integrated ensemble considers combinations of two or more from a total of 11 results, the number of such combinations is 1953, which is the total number of combinations minus those from the BERT- and feature-based ensembles.

“Integrated ensemble” refers to the aggregation of all results obtained from various aspects. The F1 score statistics for the integrated ensembles are presented in Table 4, where the top 50 results correspond to the integrated ensemble. For Corpus A, the highest F1 score was 1.00; for Corpus B, it was 0.96. In Corpus A, this score was 19 points higher than that of the single model with features; in Corpus B, it was 13.7 points higher than the maximum score of the single model, confirming the effectiveness of the integrated ensemble. Additionally, the integrated ensemble F1 scores in both corpora improved by one and two points, respectively, compared to (i) the ensemble of BERT results combined with a single feature and classifier, and (ii) the ensemble of a single BERT result combined with feature and classifier outputs. The results for the weighted ensemble were nearly identical. For comparison, the results of both ensemble methods—Ensemble One Feature & Classifiers with BERTs and Ensemble One BERT and Features & Classifiers—were also computed and summarized in Table 4. The proposed method achieved a significantly higher score than these ensemble approaches.

To evaluate the improvement of the proposed method based on the mean values presented in Table 4, we conducted Welch’s two-sample t-tests and compared the proposed method (I) with four baseline approaches: Ensemble BERTs (B), Ensemble Features and Classifiers (E), Ensemble One Feature & Classifiers with BERTs (G), and Ensemble One BERT and Features & Classifiers (H). The results of Welch’s two-sample t-tests for Corpus A are as follows: I vs. B (*p* = 0.0001, Cohen’s d = 0.88), I vs. E (*p* < 2.2 × 10^−16^, Cohen’s d = 4.168), I vs. G (*p* = 0.017, Cohen’s d = 4.546), and I vs. H (*p* = 3.2 × 10^−7^, Cohen’s d = 1.202). For Corpus B are as follows: I vs. B (*p* = 4.2 × 10^−15^, Cohen’s d = 3. 232), I vs. E (*p* < 2.2 × 10^−16^, Cohen’s d = 3. 631), I vs. G (*p* = 0.012, Cohen’s d = 4.939), and I vs. H (*p* = 3.3 × 10^−15^, Cohen’s d = 2.718).

All pairwise comparisons, except I vs. G in both corpora, showed statistically significant differences (*p* < 0.001). For the I vs. G comparison, the *p*-values were < 0.02 in both corpora, which are below the standard 0.05 significance level.

**Figure 3:**
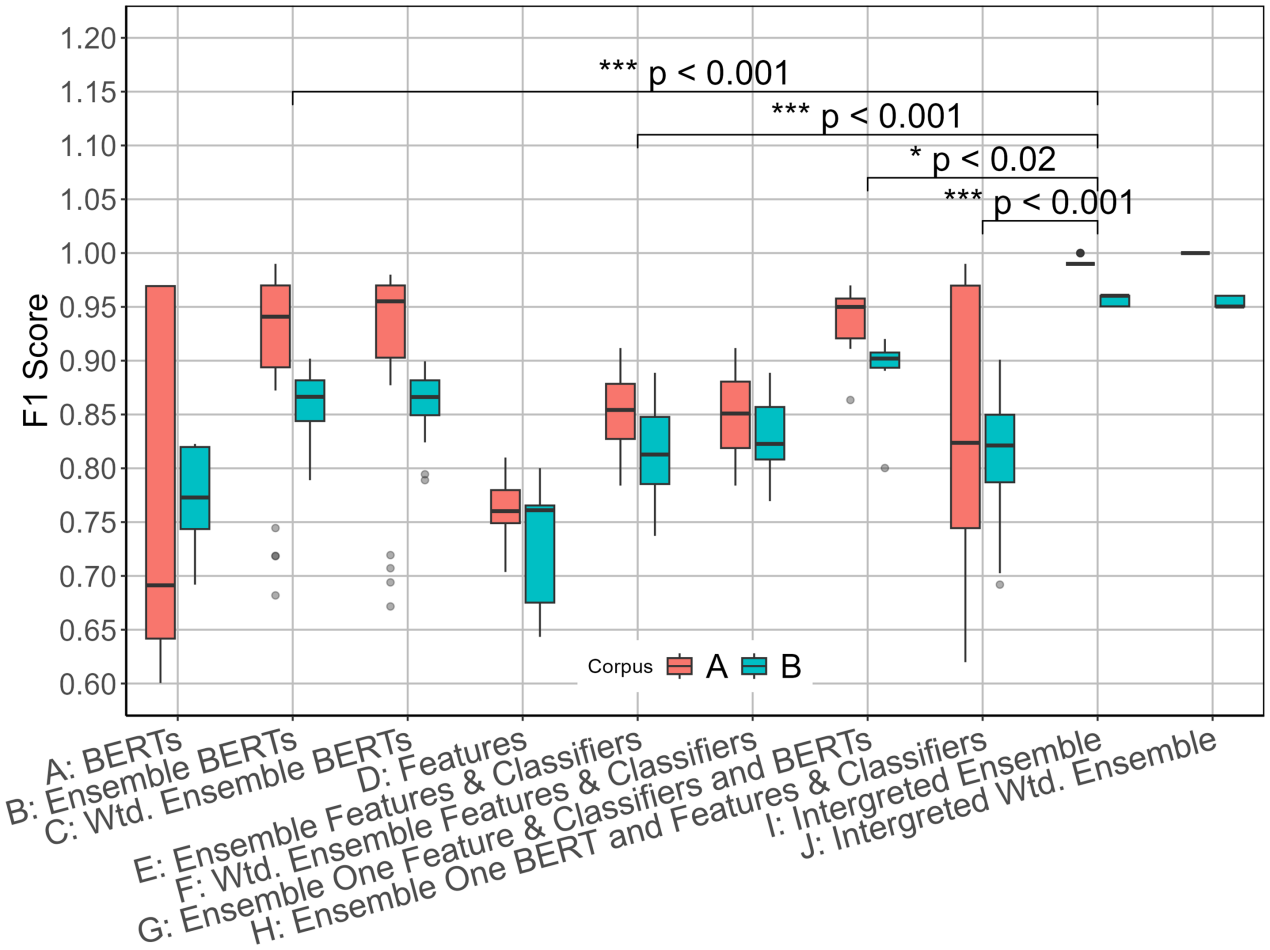
Box plot of F1 scores for both corpora.

Figure 3 presents box plots of F1 scores for both corpora. The top 50 results are shown for methods with F1 scores greater than 0.50. The integrated ensemble (I) demonstrates significantly higher F1 scores and substantially lower score variance compared to the baseline methods, as evidenced by the box plot distributions.

To facilitate the discussion of ensemble combinations, the top 10 integrative ensembles are listed in Table 5. These top 10 BERT model combinations exhibited different trends for Corpus A and Corpus B, as discussed in Subsection 4.2. The feature and classifier combinations included either Label 3 (Ada + Phrase) or Label 6 (RF + Phrase). Char-bigram and token-unigram features share some overlapping information—for example, a two-letter token is included in the char-bigram. However, the phrase pattern differs from these two features in that it can suppress textual topics more effectively, since the content words in a phrase are masked by their POS tags. We believe this property enhances ensemble performance. The last row of Table 5 summarizes the results of the ensemble across all models. These results indicate that the ensemble scores were consistently higher than those of individual models, although they were just one point below the highest score.

## 5 Discussion

### 5.1 Performance and effectiveness of the integrated ensemble

For Corpus A, Models A and AW were included in the pre-training data. Therefore, we considered excluding these two models from the integrated ensemble for Corpus A. The highest F1 score achieved by the ensemble excluding these models was 0.92, observed in the combinations {T, De 1, 3, 5, 6}, {T, S 1, 3, 5, 6}, and {De, S 1, 3, 5}. This score is 22.9 points and 11 points higher than the best scores of the standalone BERT models (0.691) and the feature-classifier combinations (0.810), respectively. Furthermore, it is 14.7 points higher than the best score (0.773) of the BERT ensemble {De, S} excluding Models A and AW, and 0.8 points higher than the best score (0.912) of the feature-classifier ensemble.

For Corpus B, the integrated ensemble results were 13.7 and 16 points higher than the highest scores of the standalone BERT model (0.823) and the feature-classifier combinations (0.800), respectively. Additionally, the score was 5.8 and 7.1 points higher than the best results of the BERT model ensemble (0.902) and the feature-classifier ensemble (0.889), respectively. Thus, the integrated ensemble significantly outperformed the individual models on both corpora. This result also surpasses that of an ensemble of multiple BERT models with a single feature set, or a single BERT model with multiple classifiers.

To further validate the robustness of our integrated ensemble method (I), we conducted Welch’s two-sample t-tests comparing it with four baseline ensembles (B, E, G, H). The results showed that method I significantly outperformed B, E, and H (*p* < 0.001). In contrast, the comparison between I and G yielded a relatively higher, yet still statistically significant, *p*-value (*p* < 0.02, which is still below the 0.05 threshold), likely due to G’s limited sample size (n = 6). These findings, supported by both F1 scores and statistical testing, confirm that the observed performance differences are unlikely to be due to random variation.

However, incorporating all models into an ensemble does not always lead to improved performance. As shown in Table 5, the F1 score for the ensemble of all BERT models is 0.91, which is 8 points lower than the 0.99 result obtained by ensembling the best models, A and S. A similar trend was also observed with both the feature-based and integrated ensembles. In some cases, the individual characteristics of certain models may negatively impact the overall score. Nonetheless, incorporating more diverse and high-performing models has the potential to further enhance the effectiveness of the integrated ensemble.

### 5.2 Factors behind ensemble effectiveness: model diversity and author-level analysis

The ensemble effect arises from the fact that individual models learn different aspects of the data, enabling them to complement one another when combined. For example, the ensemble score of BERT models T and A—each trained on distinct pre-training datasets—was higher than that of BERT model AW, which was pre-trained on a combination of those datasets. This indicates that differences in pre-training corpora, tokenizer design (e.g., MeCab vs. SentencePiece), and architectural modifications (e.g., DeBERTa’s disentangled attention) introduce diverse inductive biases that are beneficial for ensemble diversity.

As analyzed in Sections 4.3, 4.4, and 4.5, Model S yielded the lowest score among the BERT models, while Ada + Phrase and RF + Phrase produced the lowest scores among the feature-based models. Nevertheless, these models contributed significantly to achieving the highest scores in both their respective ensembles and the integrated ensemble. These findings suggest that both individual performance and the intrinsic characteristics of a model are critical when constructing effective ensembles.

To further investigate this phenomenon, we performed an author-level error analysis based on F1 scores. Given that the number of models used—including ensemble combinations—exceeds 2,000 per corpus, we focused on the two highest-scoring ensemble cases for each corpus. As shown in Table 5, the top-performing ensemble for Corpus A was {A, S | 3, 5}, and for Corpus B, it was {T, De | 1, 2}. We analyzed the behavior of these ensembles and their constituent models across individual authors.

Table 6 presents the author-wise F1 scores for the ensemble {A, S | 3, 5} and its constituent models using Corpus A. Although Model A was pre-trained on data that included Corpus A, none of its individual author scores reached 1.0. However, the ensemble achieved perfect scores across all authors. Sasaki had the highest average score across the four models, consistently receiving top scores. In contrast, Okamoto had the lowest average score of 0.65, though the ensemble improved this to 1.0. The score variance among models for Okamoto was relatively large, possibly due to the inclusion of Corpus A in the pre-training data. The difference in average F1 scores between Sasaki and Okamoto was 27.7 points, indicating substantial variability in author-level classification performance.

**Table 6:** F1 scores by author of the integrated ensemble model {A, S|3, 5} on Corpus A.

Author’s last name	A: AozoraB	S: StockMarkB	3: Ada_Pharse	5: RF_Token	Mean (A, S, 3, 5) ± SD	Ensemble {A, S | 3, 5}
Akutagawa	0.952	0.421	0.737	0.696	0.691 ± 0.218	1.000
Izumi	0.947	0.800	0.737	0.900	0.887 ± 0.095	1.000
Kikuchi	0.889	0.737	0.900	0.737	0.775 ± 0.091	1.000
Mori	1.000	0.348	0.842	0.824	0.749 ± 0.282	1.000
Natsume	1.000	0.588	0.700	0.800	0.797 ± 0.175	1.000
Sasaki	1.000	0.870	0.909	0.909	**0.922** ± 0.055	1.000
Shimazaki	1.000	0.455	0.615	0.857	0.792 ± 0.243	1.000
Dazai	0.952	0.762	0.857	0.889	0.873 ± 0.079	1.000
Okamoto	0.952	0.400	0.588	0.632	**0.654** ± 0.229	1.000
Umino	1.000	0.625	0.588	0.857	0.835 ± 0.195	1.000
mean	0.969	0.601	0.747	0.810		1.000
SD	0.037	0.187	0.125	0.094		

Bold values represent both the highest and lowest scores.

Table 7 presents the author-wise scores for the ensemble {T, De | 1, 2} and its constituent models using Corpus B. Miyabe had the lowest average score of 0.690, which improved to 0.9 through ensembling. In contrast, Ishida had the highest average score of 0.858, which reached a perfect 1.0 with the ensemble. The difference in the average scores of the two authors across individual models was 16.8 points. While this was smaller than the difference observed in Corpus A, it was still notable. This may be attributed to the fact that Corpus B was not used in BERT pre-training and that most texts were written in the 1990s, aligning more closely with the temporal characteristics of the pre-training data for these models.

**Table 7:** F1 scores by author of the integrated ensemble model {T, De|1, 2} on Corpus B.

Author’s last name	T: TohokuB	De: DeBERa	1: Ada_Pharse	2: RF_Token	mean(T, De, 1, 2) ± sd	Ensemble {T, De | 1, 2}
Suzuki	0.737	0.800	0.870	0.727	0.783 ± 0.066	1.000
Kishi	0.522	0.800	0.900	0.737	0.740 ± 0.160	1.000
Yoshida	0.762	0.857	0.800	0.800	0.805 ± 0.039	1.000
Miyabe	0.625	0.667	0.706	0.762	**0.690** ± 0.058	0.939
Morimi	0.636	0.889	0.737	0.947	0.802 ± 0.142	0.967
Ishida	0.900	0.889	0.824	0.818	**0.858** ± 0.043	1.000
Murakami_H	0.842	0.900	0.700	0.778	0.805 ± 0.086	0.899
Murakami_R	0.842	0.842	0.737	0.636	0.764 ± 0.099	0.947
Higashino	0.727	0.783	0.667	0.778	0.739 ± 0.054	0.967
Minato	0.842	0.800	0.667	0.632	0.735 ± 0.102	0.890
Mean	0.744	0.823	0.761	0.762		0.960
SD	0.120	0.070	0.083	0.091		

Bold values represent both the highest and lowest scores.

### 5.3 Impact of pre-training data and corpus characteristics

As shown in the last row of Table 5, the score for the integrated ensemble of all models was slightly lower than the highest score achieved by any individual ensemble. Nevertheless, it still represented a substantial improvement over the best-performing single model. Specifically, compared to the highest scores achieved by individual BERT and feature-based classifiers—both unaffected by ensemble effects—the score for Corpus B improved by 12.7 points. Our proposed method also outperformed the BERT model ensemble by 3 points and the feature-based ensemble by 6.1 points.

The choice of pre-training data in BERT models has a notable impact on downstream performance. For instance, in Corpus A, Models A and AW achieved scores that were 27.8 points higher than those of other models, primarily because Corpus A was included in their pre-training data. In contrast, for Corpus B, Models T, De, and S exhibited score increases of 10.2, 13.2, and 9.2 points, respectively, compared to their performance on Corpus A. This improvement can be attributed to the temporal alignment between Corpus B—which consists of works published after 1990—and the pre-training data used for these models. Corpus A, by comparison, contains texts written before 1950, making it less representative of the linguistic patterns captured during pre-training.

In this study, the F1 score for author attribution across 10 Japanese literary authors, using texts of approximately 510 tokens, exceeded 0.96. This result is comparable to those reported in previous studies using Corpus A (Jin and Murakami, 2017) and full-length novels for feature-based classification (Liu and Jin, 2022), demonstrating the effectiveness of our approach even with shorter text segments.

### 5.4 Limitations and future directions

This study was conducted under certain constraints: we used two corpora, five BERT models, three feature sets, and two classifiers. Our primary focus was to evaluate the effectiveness of the integrated ensemble, and we did not examine whether the specific BERT models, features, or classifiers employed were optimal choices. Although ensemble scores varied depending on the combination of constituent models, the overall effectiveness of the integrated ensemble remains evident.

Our analysis was limited to the first 510 tokens of each literary work, serving as a foundational step toward forensic applications that attribute authorship from short texts (Zaitsu and Jin, 2023; Zaitsu et al., 2024). This context presents further challenges, such as attribution from even shorter texts and understanding how text length affects classification accuracy. Meanwhile, authorial stylistic indicators are distributed throughout an entire text, and Tanaka et al. (2020) showed that BERT may not sufficiently capture these signals within a 510-token window. To address this, we plan to explore chunk-wise ensembling, which aims to improve performance on longer texts while balancing computational efficiency. This method involves dividing each text into non-overlapping 510-token blocks, extracting BERT outputs for each chunk, and aggregating predictions via majority voting. We also found that weighted ensembling—using model scores as weights—did not outperform the unweighted approach. These findings suggest that future research is needed to refine ensemble strategies and optimize model selection.

## 6 Conclusion

As the number of classification models increases, so does the need to apply them effectively to a AA. To address this, we examined the effectiveness of an integrative ensemble method that combines BERT-based and feature-based approaches in a small-sample AA task. Additionally, we analyzed the impact of BERT pre-training data on task performance, as well as the influence of individual models on ensemble outcomes. The corpora consist of two sets of self-generated literary works. For the integrated ensemble, we used five BERT models, three types of features, and two classifiers. A summary of the results is presented below:

- BERT proved more effective than traditional feature-based classifiers for AA in short literary works, demonstrating its utility even in small-sample scenarios.
- Both BERT-based and feature-based classifier ensembles outperformed their standalone counterparts, with the proposed integrated ensemble method achieving even higher scores. Notably, when applied to a corpus excluded from the pre-training data, the integrated ensemble elevated the F1 score from 0.823 to 0.96—an improvement of approximately 14 points—surpassing the performance of the best individual model. It achieved the highest score among all evaluated approaches, including standalone models and conventional ensemble techniques, with a statistically significant margin (*p* < 0.012), underscoring the robustness of the result.
- We confirmed that the pre-training data used for BERT significantly impacts task performance. Furthermore, in ensemble learning, individual models influence final results not only through their performance but also through the diversity of their inherent characteristics, highlighting the importance of considering both factors in model selection.

These findings highlight the critical role of model diversity and pre-training data in ensemble learning, and propose effective strategies for harnessing the growing heterogeneity of classification models. The insights and empirical results presented herein extend beyond authorship attribution in short literary texts, offering practical relevance to forensic analyses of brief communications and the detection of machine-generated content produced by large language models. Moreover, as agent-based AI systems—comprising multi-expert frameworks, multi-agent architectures, and LLM-driven agents—gain increasing prominence in addressing complex tasks (Chen et al., 2024; Tran et al., 2025), the conceptual contributions and methodological advances of this study provide valuable perspectives that may inform and enhance future research in this domain.

## Data Availability

The datasets presented in this study can be found in online repositories. The names of the repository/repositories and accession number(s) can be found at: https://github.com/mining-jin/Ensemble.

## Footnotes

^1^ https://taku910.github.io/mecab/

^2^ https://clrd.ninjal.ac.jp/unidic/download.html#unidic_bccwj

^3^ https://taku910.github.io/cabocha/

^4^https://huggingface.co/tohoku-nlp/bert-base-japanese-v2 (MeCab + WordPiece).

^5^ https://github.com/akirakubo/bert-japanese-aozora

^6^ https://huggingface.co/ku-nlp/deberta-v2-base-japanese

^7^ https://huggingface.co/stockmark/bart-base-japanese-news

^8^The dataset and computational source code for this study are available in the following GitHub repository https://github.com/mining-jin/Ensemble.

^9^ https://a3hsn.org/nlptools/
